# Functional network activation during a subtraction task

**DOI:** 10.1038/s41598-026-53617-x

**Published:** 2026-06-02

**Authors:** Anna Hutber, Suraiya Husain, Luis Diambra, Vasiliki Tsirka, Alberto Capurro

**Affiliations:** 1 Blizard Institute, Neuroscience, Surgery and Trauma, Queen Mary University of London, London, UK; 2 Universidad Nacional de la Plata, La Plata, Argentina; 3 CONICET, La Plata, Argentina; 4 Department of Neurosciences, Royal London Hospital, Barts Health NHS Trust, London, UK

**Keywords:** EEG, Spectral power, Mutual information, Functional connectivity, Mental arithmetic, Fronto-parietal, Neurology, Neuroscience

## Abstract

Arithmetic processing recruits fronto-parietal and temporal networks with frequency-specific dynamics modulated by operation and complexity. However, spectral and connectivity dynamics underlying individual differences in arithmetic performance remain poorly characterised. Here, we quantify spectral power and mutual information (MI) during serial subtraction to examine task performance correlates. Beta power in the left frontal lobe and delta power in the right temporal region were positively correlated with arithmetic score. MI between the left frontal and right temporal lobes in the beta band, as well as between the right frontal and posterior regions in beta and gamma bands, showed negative correlations with arithmetic score. In the alpha band, MI between bilateral frontal and left temporal regions was positively correlated with arithmetic score, whereas MI between the left frontal and right central derivations was negatively correlated. Overall, increased left frontal activation coupled with interhemispheric functional disconnection from contralateral posterior regions in the beta band was associated with higher arithmetic score, suggesting that selective activation and segregation from task-irrelevant activity are associated with efficient working memory and arithmetic processing. Additionally, MI between the left temporal and frontal regions in the alpha band was positively associated with improved performance, supporting attention and default mode network modulation as correlates of arithmetic task performance.

**Supplementary Information:**

The online version contains supplementary material available at 10.1038/s41598-026-53617-x.

## Introduction

Arithmetic tasks recruit a distributed fronto-parietal-temporal network that is essential for executive function, numerical representation and symbol processing^1,2^. Within this broader system, frontal regions such as the dorsolateral prefrontal cortex (DLPFC) and superior frontal gyri orchestrate executive control and working memory, alongside the dorsal anterior cingulate cortex as a critical hub for top-down cognitive control^3–7^. The intraparietal sulcus (IPS) also contributes to quantity and numerical semantics^8,9^, whilst the inferior temporal gyrus (ITG) is implicated in number symbol recognition^10^. This network overlaps with, but remains distinct from, language and visuospatial systems, including verbal number coding and fact retrieval coordinated by the left angular gyrus (AG)^11,12^, and number symbol recognition subserved by the inferior occipitotemporal cortex^13,14^. Electroencephalography (EEG) studies complement this framework by capturing the frequency-specific dynamics of arithmetic processing. Prior work has linked frontal delta/theta power to working memory demands^15^, left prefrontal-parietal alpha, beta and gamma power to information maintenance and manipulation in complex numerical tasks^16^, and theta and alpha activity for fact retrieval and procedural calculations, respectively^17^. Alpha rhythms are demonstrated to coordinate attention more broadly, through default mode network (DMN) suppression and dorsal attention network engagement^18,19^.

Functional network recruitment also varies systematically by operation and complexity^20^. Superior and inferior parietal regions, with the IPS belonging to the superior parietal lobule (SPL) and the AG to the inferior parietal lobule, encode distributed representations of subtraction, multiplication, division, and addition through differential retrieval, calculation, and inversion processes^21^. It is broadly accepted that procedural calculation (i.e., subtraction, addition) is associated with bilateral fronto-parietal engagement centred on the IPS, reflecting the demands of quantity manipulation and working memory^8^. In contrast, arithmetic fact retrieval (i.e., simple multiplication) has been primarily supported by a left-hemispheric network centred on the AG, evidenced by both single-case and patient group studies^13,22^. Although neuroimaging studies have also frequently reported bilateral parietal activation for multiplication^23^, evidence implicating the right IPS, right SPL and inferior parietal lobule, remains mixed^24–26^. Problem complexity also modulates IPS activation for two- versus one-digit problems, reflecting visuospatial working memory demands^27^. EEG extends these patterns to frequency dynamics: single-digit addition and multiplication showing operation-specific patterns, with fronto-central theta amplitude/synchronisation for addition (spatial operands) and centro-parietal alpha inter-trial phase coherence for multiplication (long-term knowledge)^28^. Frontal and occipital delta also vary with single- to multi-digit addition difficulty^29^.

Taken together, these operation- and complexity-dependent activation patterns raise questions about the neural signatures of effective arithmetic performance. Professional mathematicians have been shown to exhibit stronger bilateral IPS, ITG, and prefrontal activation across analysis, algebra, topology, and geometry compared to humanities specialists^30^. High performers in two-digit addition and subtraction tasks also demonstrate increased left inferior frontal gyrus (IFG) activation for carry and borrowing operations^31,^ while low-ability individuals display reduced superior temporal gyrus (STG), IFG, and left AG activation compared to high-ability counterparts^22,32^. However, the relationship between arithmetic tasks and ability is not simply linear. Most analyses employ categorical comparisons (high vs. low performers, mathematicians vs. controls) rather than continuous correlations across the full ability spectrum. Continuous information-theoretic approaches such as mutual information (MI) address this limitation, capturing both linear/non-linear dependencies and leveraging full data variability to identify network patterns scaling incrementally with arithmetic ability.

Although prior studies link fronto-parietal-temporal activations to arithmetic performance via categorical group contrasts, continuous correlations of frequency-specific EEG power and connectivity with performance remain largely unexamined. Here we analyse spectral power and MI from EEG during serial subtraction^33^, a complex task requiring repeated borrowing, continuous result updating, and sustained working memory and attention. Our primary exploratory aims are: (1) to confirm fronto-parietal modulation and posterior alpha suppression during arithmetic processing, and (2) to test continuous correlations of frequency-specific power and connectivity with performance, expecting enhanced fronto-parietal engagement in higher performers.

## Methods

### Data source and participants

EEG data were sourced from the PhysioNet database^34^, originally collected by Zyma et al. (2019)^33^ with approval from the Bioethics Commission of the Educational and Scientific “Institute of Biology and Medicine,” Taras Shevchenko National University of Kyiv, in accordance with the Declaration of Helsinki. The dataset comprised recordings from 66 participants initially, reduced to 36 right-handed students (9 males, 27 females; mean age 18.25 ± 4.5 years, range 17–26) at the University of Kyiv after excluding cases with excessive oculographic/myographic artefacts. All participants provided written informed consent and reported normal/corrected-to-normal visual acuity, colour vision, and no learning disabilities, psychiatric/neurological conditions, or substance addiction.

### Experimental task

Participants were seated comfortably in a darkened, sound-attenuated room with eyes closed. Following a three-minute adaptation period, three minutes of resting-state EEG were recorded. EEG recording was limited to the first minute of the subsequent 4-minute mental serial subtraction task, which involved auditory presentation of a 4-digit minuend and 2-digit subtrahend (e.g., 4753-17, 3141-42). Subtractions were completed mentally without overt movement or subvocalisation, followed by verbal response. Performance was quantified based on operations completed (range 1–34.59.59) and accuracy (reported answer as exact multiple of subtrahend). Task difficulty varied by presentation rate, operations per unit time, and problem characteristics. The original study classified participants as “good counters” (mean 21 operations, SD 7.4) versus “poor counters” (mean 7, SD 3.6), using an arbitrary score threshold of 10 for classification. A histogram of arithmetic scores revealed a flat distribution without bimodal clustering (Supplementary File S1), providing low support for categorical divisions; therefore, these groups were not used for this analysis.

### EEG recordings

EEG was recorded using a Neurocom monopolar 23-channel system (Ukraine X AI-MEDICA) with electrodes placed according to the 10–20 system in referential montage, with linked earlobes (A1-A2) as the reference. Sampling rate was 500 Hz and signals bandpass-filtered 1–45 Hz. Nineteen channels were analysed (Fp1, Fp2, F3, F4, F7, F8, T3, T4, C3, C4, T5, T6, P3, P4, O1, O2, Fz, Cz, Pz). A qualified neurophysiologist performed visual inspection for myographic/oculomotor artefacts beyond original documentation^33^. No further artifact rejection or quantitative criteria were applied, given controlled recording conditions (eyes closed, relaxation instructions).

### Data analysis

**Fig. 1:**
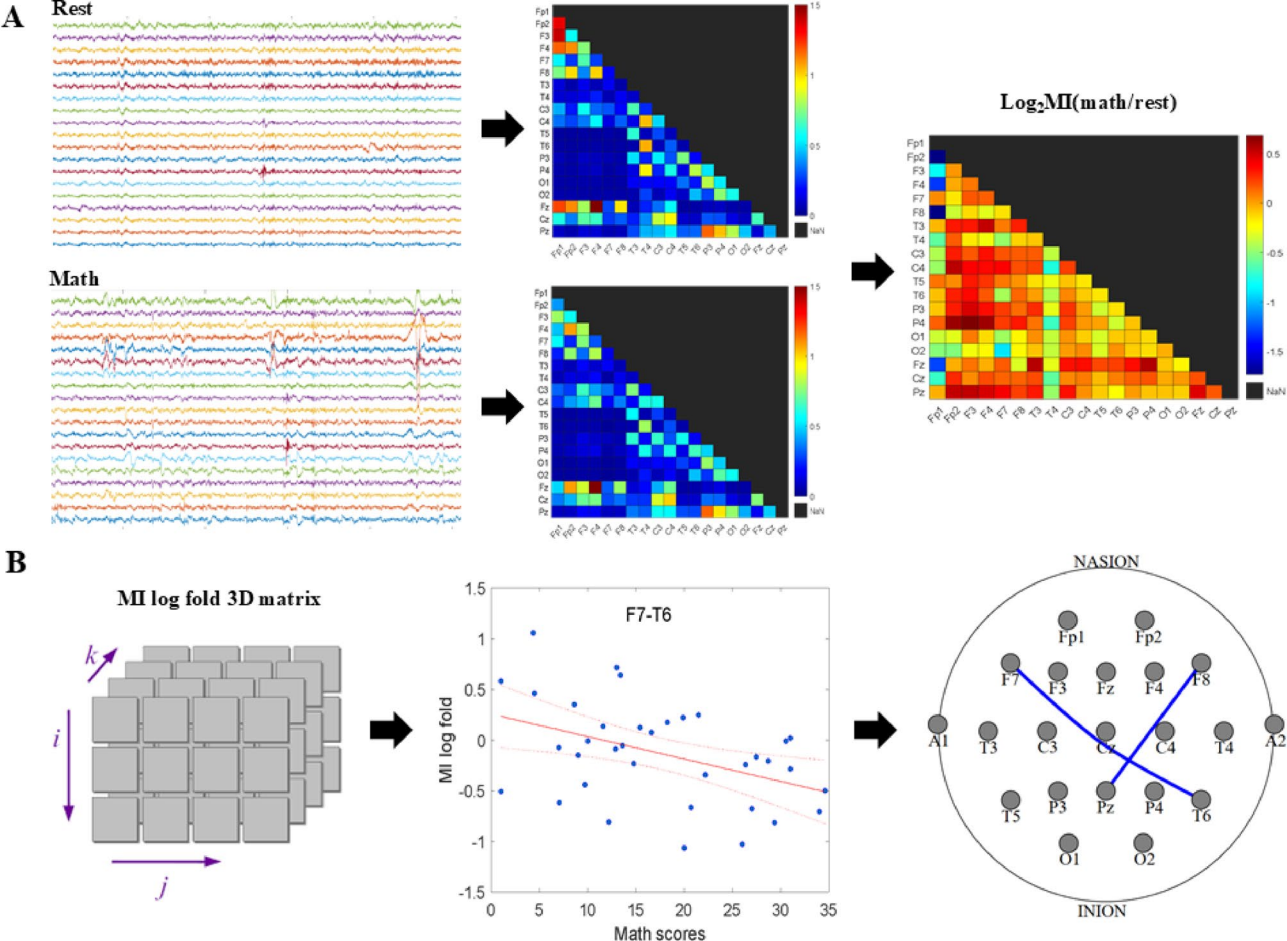
Methodological summary of EEG connectivity correlation with arithmetic performance. (**A**) Mutual information (MI) matrices were computed for rest and arithmetic conditions across 19 EEG channels (*n* = 36). Log₂(arithmetic/rest) ratios show task-related connectivity changes (warm colours = increased during arithmetic, cool colours = decreased). (**B**) Correlation analysis between MI changes and arithmetic scores identify significant electrode pairs, with F7-T6 connection shown as example. Topographic map displays spatial distribution of significant correlations, indicated by connecting lines (red for positive, blue for negative).

EEG was recorded continuously for 3 minutes during resting-state and the first 60 seconds of the 4-minute serial subtraction task (Fig. 1). Analysis focused on the first minute of each condition, using rest as baseline to capture task-induced changes while matching durations. Signals were bandpass filtered (MATLAB’s *filtfilt* function, steepness 0.8) into standard bands: delta (1–4 Hz), theta (4–8 Hz), alpha (8–13 Hz), beta (13–30 Hz), gamma (30–45 Hz).

Spectral power (SP) quantified activation patterns across bands for each participant (*n* = 36). SP was computed for all 19 channels using MATLAB’s *fft* function. Log fold change in SP (arithmetic task vs. rest) was calculated per channel (1 = doubling, −1 = halving), then averaged across participants (Fig. 1). Planar head maps displayed significant Spearman correlations with arithmetic scores, combining all bands due to uniformly positive effects.

MI (Eq. 1) measured statistical dependence between channel pairs^35,36^. MI (bits) used bivariate histogram estimation (50 bins) following established MATLAB procedures^37^. Per subject, MI formed 19 × 19 matrices for arithmetic and rest states; arithmetic/rest ratios underwent log2 transformation for fold change. These yielded 3D matrices (19 × 19 × 36 subjects), analysing the upper triangle (171 unique pairs, excluding diagonal). Each hyper-column represented log fold MI for one channel pair across frequency bands.

1 $$\:MI\left(X;Y\right)=\sum\:_{y\in\:Y}\sum\:_{x\in\:X}p\left(x,y\right){log}_{2}\left(\frac{p\left(x,y\right)}{p\left(x\right)p\left(y\right)}\right)$$

### Statistical analysis

The original study’s classification of participants as “good” (*n* = 26) and “poor” (*n* = 10) counters was not used in this study. Instead, performance was treated continuously, with Spearman correlations computed between scores and both spectral power (SP; 19 channels) and MI (171 channel pairs) using MATLAB’s *corr* function.

False discovery rate (FDR) correction^38,39^ yielded no significant results. Given the exploratory nature of this analysis, high EEG inter-individual variability, and limited sample size (*n* = 36), uncorrected *p*-values were reported alongside effect sizes (|ρ| ≥ 0.25), prioritising hypothesis generation over confirmatory inference^40^.

Linear regression slopes (|ρ|) and uncorrected Spearman *p*-values appear in Table 1 (SP) and 2 (MI). Thresholds were *p* ≤ 0.05 (significant) and *p* ≤ 0.1 (trends). Scatterplots for *p* < 0.05 are shown in Supplementary File S2. Topographic maps display SP correlations (beta=yellow, delta=blue) and MI connections (red=positive, blue=negative).

Post-hoc power analysis employed Fisher’s z-transformation on Spearman correlation *p*-values (using MATLAB’s sampsizepwr function). Tables 1 and 2 report achieved power for *n* = 36 alongside required sample sizes to achieve the same *p*-values at 80% power (two-tailed).

## Results

**Fig. 2:**
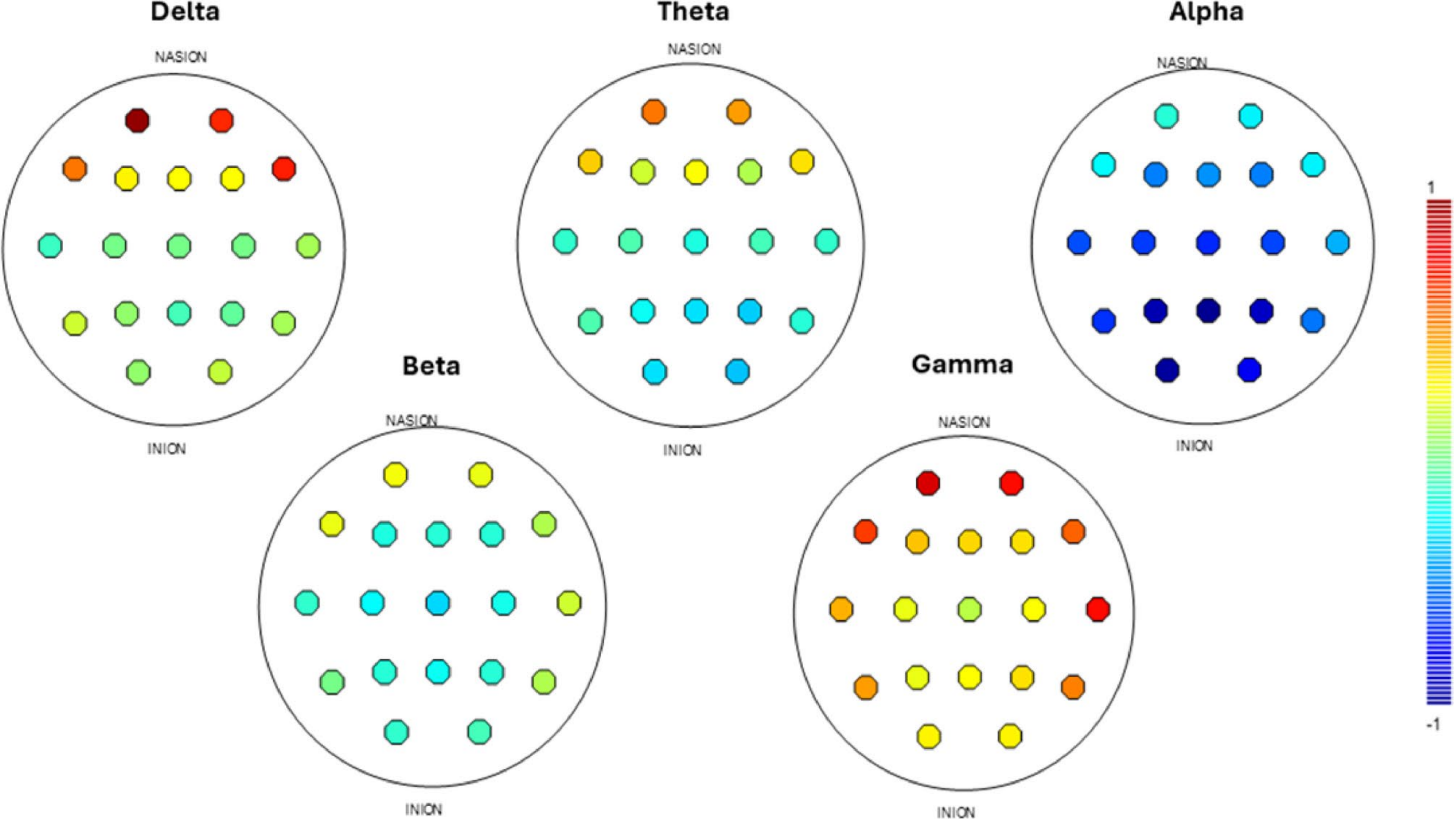
Planar head representations showing log fold arithmetic/rest mean values of spectral power in each frequency band (*N* = 36). A value of 1 indicates duplication of spectral power during arithmetic, whilst a value of −1 indicates halving.

Serial subtraction elicited distinct frequency-specific activation patterns across cortical regions. Frontal regions showed increased spectral power in delta (1–4 Hz), theta (4–8 Hz), and beta (13–30 Hz) bands during arithmetic processing, with the most pronounced and widespread activation occurring in the gamma (30–45 Hz) band (Fig. 2). In contrast, alpha (8–13 Hz) power showed substantial suppression during the task, predominantly in posterior regions. These patterns are consistent with established literature on arithmetic cognition and confirm the quality of the dataset.

**Fig. 3:**
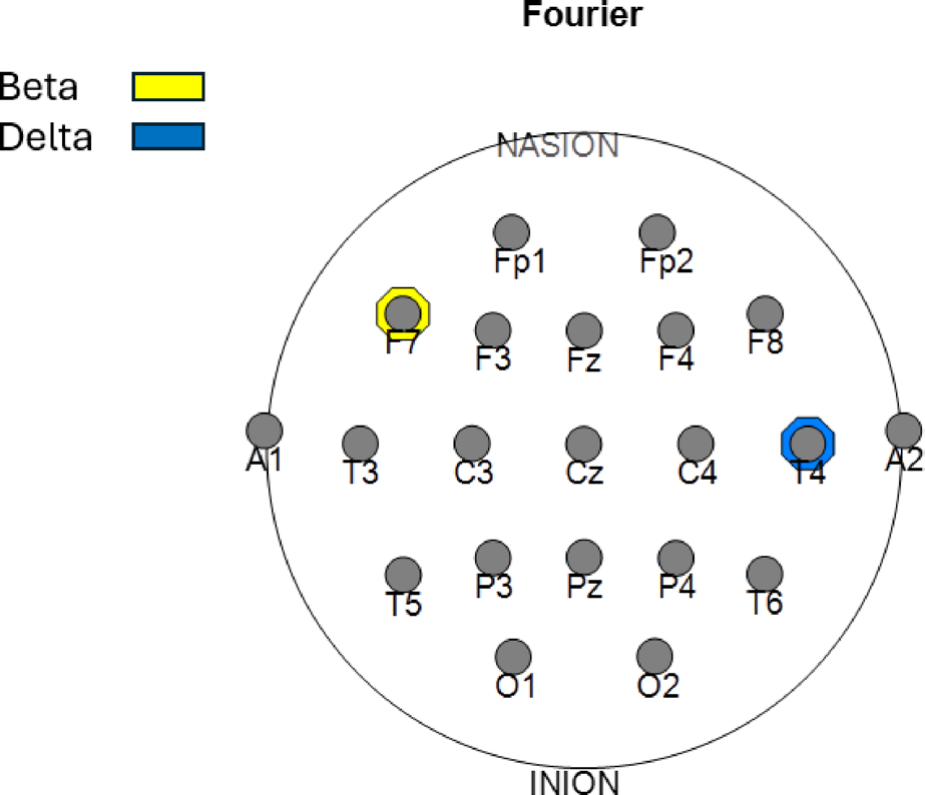
Planar head representation showing the channels with significant positive Spearman correlation between mean log fold arithmetic/rest of spectral power and the arithmetic scores (*p* < 0.05, *N* = 36). The frequency bands are indicated with a colour code (yellow=beta, blue=delta). Positive correlations were found only in the beta and delta bands. No significant negative correlations were found.

Arithmetic performance correlated significantly with spectral power changes in two key regions (Fig. 3). An increase in beta power in the left frontal cortex (F7) demonstrated a positive correlation with arithmetic score (*p* < 0.05), consistent with dominant hemisphere engagement in right-handed participants and previous reports of frontal beta involvement in arithmetic calculation. Additionally, delta power increases in the right temporal cortex (T4) were positively correlated with score (*p* < 0.05). Marginally significant positive correlations were also observed at T5, T6, and Fz electrodes (*p* < 0.1; Table 1).

**Fig. 4:**
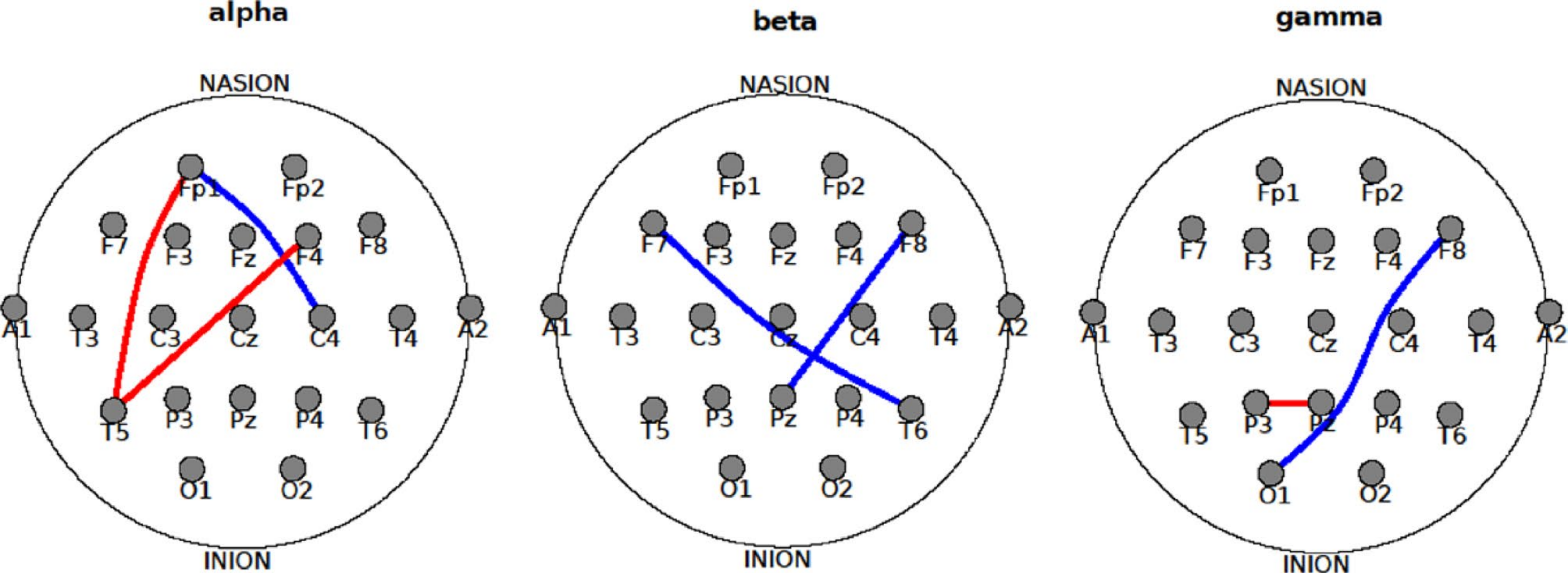
Planar head representations of functional connections with positive (red traces) and negative (blue traces) slopes (|ρ|) in the linear regressions reaching significance (*p* < 0.05) in the Spearman correlation of mutual information (MI) with the arithmetic scores. Each head corresponds to a different frequency band (alpha, beta, gamma).

Functional connectivity analysis revealed that performance was associated with specific patterns of cortical decoupling. Reduced beta-band connectivity between frontal and posterior regions - specifically F7-T6 and F8-Pz connections - correlated significantly with higher arithmetic score (*p* < 0.05). This suggests that a disconnection between frontal and posterior areas in the beta band is associated with better task performance. A similar pattern emerged in the gamma band, where frontal-posterior decoupling (F8-O1) also predicted better performance, whilst enhanced coupling within the posterior left hemisphere showed positive correlations with arithmetic score (Fig. 4).

Alpha-band connectivity showed a distinct pattern in the faster beta and gamma frequency bands. Positive correlations with arithmetic performance were observed for connections between posterior right temporal cortex (T5) and bilateral frontal regions (Fp1 and F4), suggesting that enhanced alpha coupling between temporal and frontal areas may facilitate arithmetic processing (Fig. 4). Conversely, alpha connectivity between left frontal (Fp1) and right central (C4) regions showed a negative correlation with performance, indicating that reduced frontal-central coupling in the alpha band is associated with better arithmetic abilities. No significant correlations were found between connectivity measures and arithmetic scores in the slower delta and theta frequency bands. All cases meeting trend-level significance (*p* < 0.1) are listed in Table 2, whilst the correlation scatter plots of cases reaching *p* < 0.05 are presented in the figure S1.

Correlations meeting statistical significance (*p* ≤ 0.05) demonstrated medium effect sizes, with Spearman correlation coefficients ranging from 0.33 to 0.41 in absolute value (Tables 1 and 2). These effect sizes indicate meaningful relationships between neural connectivity patterns and arithmetic performance in the context of neurobiological studies. The monotonic nature of these relationships is illustrated in scatter plots (Supplementary File S2), demonstrating slopes distinct from horizontal lines. Post-hoc power analysis using Fisher’s z-transformation revealed that our sample of 36 participants provided approximately 50% statistical power (*α* = 0.05) to detect true correlations, whereas achieving adequate power of 80% would require sample sizes ranging from 56 to 79 participants depending on effect size (|ρ|=0.2–0.4; Tables 1 and 2).

**Table 1:** Linear regression slopes (|ρ|) and p values in Spearman correlation between spectral power log fold and arithmetic scores, showing cases reaching *p* < 0.1. Cases reaching *p* < 0.05 are highlighted in yellow. Power achieved for *n* = 36 and number of subjects required to achieve power of 0.8 are reported in columns 5 and 6.

Channel	Frequency	|ρ|	*p* value	Power	*N* for power = 0.8
F7	Beta	0.376	0.024	0.505	65
T4	Delta	0.483	0.003	0.517	56
T5	Delta	0.317	0.059	0.501	72
T6	Alpha	0.279	0.099	0.499	79
Fz	Delta	0.281	0.097	0.500	79

**Table 2:** Linear regression slopes (|ρ|) and p values in Spearman correlation between mutual information (MI) and arithmetic scores, showing channel combinations reaching *p* < 0.1. Cases reaching *p* < 0.05 are highlighted in yellow. Power achieved for *n* = 36 and number of subjects required to achieve power of 0.8 with the same p-value are reported in columns 5 and 6.

Channel 1	Channel 2	|ρ|	*p* value	Power	*N* for power = 0.8
**Delta**					
F8	Fp1	0.297	0.079	0.500	76
T3	F3	− 0.280	0.099	0.499	79
P3	C3	0.327	0.052	0.501	71
**Theta**					
P4	P3	0.289	0.088	0.500	77
Pz	O1	− 0.321	0.057	0.501	72
**Alpha**					
C4	Fp1	− 0.364	0.029	0.504	66
T5	Fp1	0.412	0.013	0.508	62
T5	F4	0.397	0.016	0.506	63
T5	T4	0.281	0.097	0.500	79
**Beta**					
T3	F8	− 0.315	0.061	0.501	73
T6	F7	− 0.397	0.016	0.506	63
P3	F8	− 0.301	0.075	0.500	75
P4	Fp1	− 0.281	0.096	0.500	78
O2	T5	0.279	0.100	0.499	79
Fz	F7	− 0.286	0.090	0.500	78
Cz	F7	− 0.293	0.083	0.500	76
Cz	T6	− 0.280	0.098	0.499	79
Pz	Fp1	− 0.311	0.065	0.501	73
Pz	F8	− 0.330	0.049	0.502	70
**Gamma**					
T6	C4	− 0.315	0.061	0.501	73
P3	T4	0.294	0.082	0.500	76
P3	C3	0.307	0.068	0.500	74
P3	C4	0.283	0.095	0.500	78
P3	T5	0.301	0.074	0.500	75
O1	F8	− 0.361	0.031	0.503	67
O1	T6	− 0.280	0.098	0.500	79
O1	P3	0.297	0.079	0.500	76
Pz	P3	0.382	0.021	0.505	64

## Discussion

We employed a within-subjects differential approach using log-fold change (arithmetic task divided by resting state) to evaluate both activation patterns and functional connectivity changes during a serial subtraction task. Despite a small sample size, correlations demonstrated medium effect sizes ranging from 0.3 to 0.5 (Tables 1 and 2), which represent meaningful associations within biological EEG studies accounting for substantial inter-individual variability.

### Spectral power correlates of arithmetic performance

Higher arithmetic performance correlated with increased spectral power in left frontal and bilateral temporal regions. Left frontal beta power (F7) showed a signification association with performance (Fig. 3), consistent with executive control and active maintenance of task-relevant representations in working memory^41,42^. Bilateral temporal activations in the delta band (T4 right, T5 left) were also significantly positively associated with performance. These electrodes overlie regions near the angular gyri, previously implicated in arithmetic fact retrieval and verbal arithmetic strategies^23,43^. Delta activation in these regions may reflect sustained inhibition of task-irrelevant sensory processing to facilitate internal concentration on numerical representations^44,45^, whilst the bilateral temporal engagement likely supports lexical-semantic processing during multi-digit arithmetic^46^. The contrast between frontal beta and temporal delta activation patterns reflects distinct functional roles: frontal beta supports active maintenance of working memory contents and interference prevention, whereas temporal delta facilitates sustained internal focus on numerical representations through inhibitory mechanisms. An additional interpretation is that involvement of fact-retrieval regions may reflect the decomposition of complex arithmetic problems (e.g., those involving carry or borrow operations) into smaller components solved and maintained in working memory^47^. Under this account, problem solving would engage both procedural processes and arithmetic fact retrieval, consistent with our results.

### Functional connectivity: frequency-specific patterns

Connectivity patterns showed striking frequency specificity. In beta and gamma bands, better performance was associated with reduced connectivity (functional disconnection) between frontal and posterior regions. The most significant finding was decreased beta-band connectivity between left frontal (F7) and right temporal (T6) regions (Fig. 4), with similar patterns observed for right frontal to central parietal (F8-Pz, beta) and right frontal to occipital (F8-O1, gamma) connections. This interhemispheric disconnection, also observed during complex versus simple arithmetic by others^16^, likely reflects suppression of task-irrelevant posterior processing to protect arithmetic representations in prefrontal working memory^48,49^. Notably, this finding is consistent with evidence of fronto-parietal hyperconnectivity in developmental dyscalculia^50^, where excessive connectivity is associated with poorer arithmetic performance.

Additionally, a significant functional connection between electrodes P3 and Pz in the gamma band (Fig. 4) was observed, a region often regarded as central to mental arithmetic within the fronto-parietal network. This association was not observed bilaterally at P4, likely attributable to inter-individual variability and limited sample size. These findings align with left-dominant or bilateral activation during subtraction tasks observed in neuroimaging studies^21^.

Conversely, alpha-band showed significantly increased connectivity between left temporal (T5) and bilateral frontal regions (Fp1, F4), correlating with better performance (Fig. 4). This left-lateralised frontotemporal coupling, combined with posterior alpha suppression (dorsal alpha blocking), suggests higher-performing individuals enhance selective routing of task-relevant numerical information from temporal to frontal regions whilst simultaneously inhibiting competing DMN activity^51,52^. These frequency bands thus serve complementary roles: alpha facilitates selective information routing, whilst beta and gamma decoupling protects working memory from interference^41,42^. Notably, delta and theta bands showed robust task-related activation but no significant performance correlations, suggesting slower oscillations index general cognitive engagement rather than individual performance differences within non-clinical samples.

### Emotional regulation and arithmetic performance

The demanding nature of the serial subtraction task raises the question of whether our results could reflect stress-related responses due to mathematical anxiety entangled with arithmetic processing. Alpha rhythms coordinate attention via DMN suppression and dorsal attention network engagement, and mathematics anxiety has been shown to disrupt this by impairing DMN deactivation, permitting self-referential and emotional processing to compete with task-relevant cognitive resources^53^. Previous analysis of the electrocardiography (ECG) data included in this dataset^54^ reported heart rate increases of approximately 10 beats per minute during the task (from 75 to 85–88 bpm), with comparable changes in both good and poor performers. We confirmed this finding, together with the observation that heart rate remained stable during the control period, with no differences between the first and third minutes. As both heart rate values fall within normal resting range and represent only a modest increase, these findings suggest moderate rather than severe stress during the task. Crucially, the comparable heart rate increases between good and poor performers argue against a simple stress-performance correlation driving our neural findings. The observed activations in the left dorsolateral prefrontal cortex and dorsal attention networks are consistent with both arithmetic processing and emotional regulation literature, and these systems likely operate in concert during demanding arithmetic tasks. Whilst we cannot definitively isolate domain-specific numerical effects from task-general attentional, working memory and regulatory processes, the performance correlations observed and their consistency with established literature on arithmetic processing suggest our findings reflect genuine task-related neural modulation. We therefore frame our findings as reflecting modulation by arithmetic task demands, whilst acknowledging that individual differences in emotion regulation and cognitive control may also contribute.

### Methodological comparison with other analyses of this dataset

Several studies have examined this dataset^55–57^, with findings broadly consistent regarding task-related activation patterns: increased frontal beta and gamma activation during subtraction and elevated dorsal alpha power at rest^58–60^. However, direct comparison is limited by methodological heterogeneity, including differences in referencing schemes, frequency band definitions, epoch segmentation, and connectivity algorithms - alongside the near-universal use of categorical performance classifications rather than continuous correlations. Most prior studies dichotomised participants into “good” versus “poor” performers^54,55,59,61^, thereby discarding graded performance information. For instance, Nadiya et al. (2023)^59^ used spectral edge frequency in beta and gamma bands as a categorical classifier to separate task conditions and group individuals by counting ability, whilst Balani et al. (2024)^62^ reported increased fronto-parietal connectivity during arithmetic versus rest (consistent with our alpha-band findings) but did not differentiate by frequency or examine continuous performance relationships. Our analysis revealed a flat performance histogram (*N* = 36) without natural clustering (Supplementary file S1), prompting a continuous approach via Spearman correlation to preserve ordinal information across the full ability spectrum rather than imposing arbitrary thresholds.

### Limitations

In this study, we used functional activation measures to assess the neural correlates of arithmetic performance in adults. Whilst these techniques can contribute to our understanding of brain-behaviour relations during demanding cognitive tasks, several limitations must be considered.

Localising sources of neural activity with low-density EEG is challenging and requires many assumptions compared to techniques with superior spatial resolution such as functional magnetic resonance imaging (fMRI), magnetoencephalography (MEG), or high-density EEG. Nevertheless, findings of longer-range connections and the absence of short-range connections are unlikely to be explained by volume conduction artefacts. Furthermore, detailed information on specific arithmetic operations (e.g., number of borrowing operations) was not reported in the original study, limiting our ability to analyse performance as a function of task-specific demands. The absence of a true non-numerical active control condition (e.g., serial letter recitation) additionally limits strictly domain-specific claims about numerical processing, though our performance correlations link EEG features directly to subtraction accuracy. We acknowledge that the observed activations in dorsal attention and parietal networks are shared among many demanding cognitive tasks.

The retrospective secondary analysis of a specific and limited sample of university students restricts the generalisability of results to individuals of different ages, educational backgrounds, or performing different cognitive tasks. Moreover, notable inter-individual variability was observed across the dataset, which is characteristic of EEG studies. Optimal strategies for multiple comparison correction in high-dimensional EEG connectivity remain under debate, with no universal consensus on the best approach for dense tests across channels, pairs, and frequency bands^63^. Whilst per-edge methods such as Bonferroni or FDR are straightforward, they tend to be overly conservative for the spatially and temporally correlated structure of EEG data, often eliminating distributed network effects and thereby increasing false negative risk. Approaches vary widely in the literature: the original authors used an adapted Bonferroni method^56^, some applied no correction on this dataset^55^, whilst others advocate for non-parametric cluster-based permutation testing as a more sensitive alternative that controls family-wise error rate at the network level, though this lacks precise localisation^64–66^. In this context, we acknowledge that our findings should be considered exploratory and require replication in larger, independent samples. To guide future research in this area, we conducted a statistical power calculation to estimate the sample size required to achieve 80% power under similar variability, as detailed in Tables 1 and 2.

## Conclusion

In summary, our results confirm previous observations of effective attention allocation through alpha modulation and fronto-parietal network activation during subtraction tasks. We extend current understanding of network modulation by identifying decoupling between left frontal and contralateral temporal regions, indicating region-specific activation with functional segregation from task-irrelevant brain activity, which is associated with improved arithmetic performance. Our findings highlight the utility of continuous, information-theoretic approaches, even with low-density EEG datasets, for studying the neural dynamics underlying numerical cognition. This study underscores the need for larger and more diverse cohorts in future research and suggests that continuous analyses may ultimately enhance both theoretical understanding and practical assessment of arithmetic ability.

## Supplementary Information

Below is the link to the electronic supplementary material.

Supplementary Material 1

Supplementary Material 2

## Data Availability

This dataset is publicly available and can be found on the Physionet database^34^, originally collected by^33^ in ‘EEG During Mental Arithmetic Tasks’.

## Abbreviations

EEG: Electroencephalography
SP: Spectral power
MI: Mutual information
