# Supplementary material for: Functional network activation during a subtraction task
Source: Sci Rep. 2026 Jun 2;16:25154. doi: 10.1038/s41598-026-53617-x (PMC13470476; doi:10.1038/s41598-026-53617-x)
Supplement: Supplementary file 1 [file 41598_2026_53617_MOESM1_ESM.docx]

*Supplementary Figure 1: Linear regressions of T4 delta power (left panel) and F7 beta power (right panel) on the arithmetic scores. Slopes (|ρ|) and p values in the Spearman's correlation are indicated in the corresponding panels.*

*Supplementary Figure 2: Linear regressions of MI with arithmetic scores for channel pairs reaching p <0.05 in the Spearman's correlation (alpha frequency band). Slopes (|ρ|) and p values are indicated in the corresponding panels. Channels pairs are specified in Y-axis labels.*

*Supplementary Figure 3: Linear regressions of MI with arithmetic scores for channel pairs reaching p<0.05 in the Spearman's correlation (beta frequency band). Slopes (|ρ|) and p values are indicated in the corresponding panels. Channels pairs are specified in Y-axis labels.*

*Supplementary Figure 4: Linear regressions of MI with arithmetic scores for channel pairs reaching p<0.05 in the Spearman's correlation (gamma frequency band). Slopes (|ρ|) and p values are indicated in the corresponding panels. Channels pairs are specified in Y-axis labels.*
