# Supplementary material for: Functional network activation during a subtraction task
Source: Sci Rep. 2026 Jun 2;16:25154. doi: 10.1038/s41598-026-53617-x (PMC13470476; doi:10.1038/s41598-026-53617-x)
Supplement: Supplementary file 2 [file 41598_2026_53617_MOESM2_ESM.docx]

*Supplementary Figure 1: Histogram of the arithmetic scores using 25 bins showing a uniform distribution.*
